# Research progress on the relationship between free fatty acid profile and type 2 diabetes complicated by coronary heart disease

**DOI:** 10.3389/fendo.2024.1503704

**Published:** 2024-12-06

**Authors:** Xiuyan Liu, Ming Gong, Na Wu

**Affiliations:** ^1^ Department of Endocrinology, Shengjing Hospital of China Medical University, Shenyang, China; ^2^ Department of Pediatrics, Shengjing Hospital of China Medical University, Shenyang, China

**Keywords:** free fatty acid profile, polyunsaturated fatty acids, coronary heart disease, type 2 diabetes, atherosclerosis

## Abstract

Patients with type 2 diabetes mellitus (T2DM) have a 2 to 3 times higher risk of cardiovascular disease compared to non-diabetic individuals, and cardiovascular disease has consistently been a leading cause of death among diabetic patients. Therefore, preventing cardiovascular disease in diabetic patients remains a significant challenge. In addition to classic indicators such as cholesterol and lipoproteins, previous studies have demonstrated that plasma level of free fatty acid (FFA) is closely related to the occurrence of atherosclerosis, particularly in T2DM patients. In recent years, with further research and advancements in testing technologies, the FFA profile has garnered widespread attention. The FFA profile includes many different types of FFAs, and changes in the plasma FFA profile and concentrations in T2DM patients may lead to the development of insulin resistance, causing damage to vascular endothelial cells and promoting the occurrence and progression of atherosclerosis. Furthermore, some FFAs have shown potential in predicting cardiovascular complications in T2DM and are associated with the severity of these complications. Here, we aim to review the changes in the FFA profile in T2DM and discuss the relationship between the FFA profile and the occurrence of vascular complications in T2DM.

## 1 Introduction

Diabetes is a chronic metabolic disorder characterized by insufficient insulin secretion and/or insulin resistance caused by environmental and genetic factors. The prevalence of diabetes is increasing year by year. There are 537 million adults worldwide living with diabetes, and by 2045, the prevalence of diabetes is expected to increase by 16%, affecting 783 million people globally (1). Diabetes is associated with many medical complications, with cardiovascular diseases remaining the leading cause of death and disability among diabetes patients, especially in those with type 2 diabetes mellitus (T2DM) (2). T2DM accounts for more than 90% of diabetes cases (3), and its most common and severe complication is coronary heart disease, which accounts for about two-thirds of deaths in T2DM patients (4, 5).

Diabetes is a group of carbohydrate metabolism disorders characterized by chronic hyperglycemia, resulting from insufficient insulin secretion, defective action, or both. Insulin deficiency can lead to various metabolic abnormalities in proteins, lipids, and carbohydrates. Coronary artery disease primarily progresses due to atherosclerosis, manifested as lesions in the intima of the arterial wall and plaque accumulation, which narrows the lumen and leads to ischemia and metabolic changes in the tissues supplied by the affected arteries. Subsequent erosion and rupture of atherosclerotic plaques can trigger thrombotic events, posing a risk of fatality. Many studies have investigated the potential link between these two chronic diseases (6–9), and the results indicate that diabetes itself can induce the formation of atherosclerotic plaques or further accelerate their development. Elevated blood glucose levels, dyslipidemia, and other metabolic changes associated with the progression of comorbidities are closely related to the pathogenesis of atherosclerosis at nearly every step of the process. In addition to classic risk factors such as cholesterol and lipoproteins, previous studies have demonstrated that level of free fatty acid (FFA) is closely associated with the occurrence of atherosclerosis, particularly in patients with T2DM.

FFA is an important energy substrate for the human body and is involved in many metabolic regulations (10). FFA mainly originates from adipose tissue, released through the breakdown of triglycerides, and is primarily carried in the blood by serum albumin (11). FFA includes saturated fatty acids (SFAs) and unsaturated fatty acids (UFAs), with the latter further divided into monounsaturated fatty acids (MUFAs) and polyunsaturated fatty acids (PUFAs). They perform different functions due to their structural differences. The FFA profile can be regarded as a collective term for different types of FFAs. An important analytical method for detecting and analyzing the FFA profile is liquid chromatography-tandem mass spectrometry (LC-MS/MS), which has advantages over traditional immunoassays and spectroscopic methods in terms of high throughput, high accuracy, high sensitivity, and high specificity. FFA level is associated with insulin resistance and the development of diabetes mellitus (12–14). Higher FFA level can predict the occurrence and severity of atherosclerotic plaques in patients with T2DM (15–19), and may also be related to the prognosis of patients with coronary heart disease (20). Previous studies on the atherosclerosis risk in diabetic patients have focused more on the total level of FFA, but there is less research on the specific composition and concentration changes of FFAs. Circulating FFA concentrations depend not only on dietary intake but also on endogenous synthesis and metabolism. Estimating individual FFA quality intake through dietary assessment is relatively difficult, and measuring circulating FFAs as biomarkers is considered more reliable compared to self-reported assessments of fatty acid intake (104). In recent years, with further research and advancements in testing technology, the FFA profile has attracted widespread attention. The FFA profile contains many different types of FFAs. The total plasma FFA level in patients with T2DM are often elevated, with the most commonly observed increase in their FFA profile being SFA (especially palmitic acid, C16:0) (21). C16:0 is the most abundant fatty acid in the human body, and its abnormal increase within cells can weaken insulin signaling, leading to the development of insulin resistance (22). Insulin resistance alters systemic lipid metabolism, resulting in high levels of triglycerides, low levels of high-density lipoprotein, and the presence of small, dense low-density lipoproteins. These lipid changes, along with endothelial dysfunction induced by abnormal insulin signaling, contribute to the formation of atherosclerotic plaques (23). Although the exact mechanisms behind the changes in blood FFA profiles in diabetic patients are not yet clear, it is believed that they may be related to changes in dietary fats and lipid metabolism (24). Some FFAs have shown potential in predicting cardiovascular complications in T2DM and are associated with the severity of these complications. Here, we aim to review the changes in the FFA profile in T2DM and discuss the relationship between the FFA profile and the occurrence of vascular complications in T2DM.

## 2 The sources and classification of FFA

Fatty acids (FAs) mainly exist in three ester forms in living organisms: triglycerides, phospholipids, and cholesterol esters. When FAs are not in ester form in plasma, they are referred to as non-esterified FAs or FFAs. FFAs are classified based on their saturation into SFAs and UFAs. SFAs mainly include stearic acid (C18:0) and palmitic acid (C16:0). UFAs are further divided into MUFAs and PUFAs based on the number of double bonds. The MUFAs present in the human body mainly include palmitoleic acid (C16:1) and oleic acid (C18:1), while the PUFAs mainly include linoleic acid (C18:2), alpha-linolenic acid (C18:3), arachidonic acid (C20:4), and docosahexaenoic acid (C22:6), each having different functions and physiological effects. Omega-3 fatty acids and omega-6 fatty acids both belong to PUFAs, and their distinction lies in the position and distance of the double bond from the methyl end of the fatty acid molecule.

SFAs mainly come from animal fats, coconut oil, and palm oil. MUFAs are primarily sourced from tea seed oil and olive oil. Long-chain omega-3 fatty acids, eicosapentaenoic acid (EPA), and docosahexaenoic acid (DHA) are found in fish oil, flaxseed oil, supplements, and concentrated pharmaceutical preparations. Omega-6 fatty acids mainly come from soybean oil, peanut oil, sesame oil, and others.

## 3 FFA profile and T2DM

### 3.1 How FFA affects insulin resistance and glucose metabolism

β-cell dysfunction and insulin resistance are fundamental characteristics of T2DM, thus studying the pathogenic mechanisms that lead to these two defects is an important avenue for researching the pathogenesis of T2DM. FFAs are closely related to both pathways. The long-term elevated glucose levels lead to glucotoxicity, which impairs the function of pancreatic β-cells. This is specifically manifested as glucose desensitization, cellular exhaustion, and chronic progressive irreversible damage to β-cells. Excessive systemic FFA and dietary lipids enter non-adipose organs such as the liver, muscle, and pancreas, accumulating in the form of ectopic fat, resulting in lipotoxicity. Toxic lipids disrupt organelles, such as mitochondria, endoplasmic reticulum, and lysosomes. Disrupted organelles release excessive reactive oxygen species (ROS) and pro-inflammatory factors, leading to systemic inflammation. Prolonged low-grade systemic inflammation can hinder insulin’s action in the insulin signaling pathway, disrupt glucose homeostasis, and lead to systemic dysregulation (25). Insulin signaling molecules are also present in β-cells, where they play a role in β-cell function. Lipotoxicity causes a weakening of the insulin signaling pathway, which can result in insulin resistance in β-cells, making it one of the important mechanisms leading to β-cell dysfunction (26). The combination of glucotoxicity and lipotoxicity, known as glucolipotoxicity, is considered particularly harmful to the quality and function of β-cells (27).However, whether the elevated levels of FFAs during the progression to diabetes are pathogenic remains unproven (28).

The harmful effects of lipotoxicity are mediated by SFAs (mainly C16:0). C16:0 is the most abundant fatty acid in the human body. An abnormal increase of C16:0 within cells, exceeding the oxidative capacity of mitochondria, may lead to the conversion into harmful complex fatty acid-derived lipids, such as diacylglycerol (DAG) and ceramides. DAG activates protein kinase C (PKC), which primarily weakens the insulin signaling pathway by phosphorylating the serine residues of insulin receptor substrate (IRS-1) (22). Ceramides act as mediators of programmed cell death and are involved in the apoptosis of pancreatic β cells. Ceramides also interfere with the expression of IRS-1 and phosphoinositide 3-kinase and protein kinase B (PKB or AKT), further weakening insulin signal transduction (29). Additionally, excessive C16:0 can impair the function of organelles (endoplasmic reticulum and mitochondria) and promote the activation of pro-inflammatory transcription factors (such as NF-kB), triggering chronic inflammation (22). UFAs can mitigate the harmful effects of SFAs to some extent by increasing the mitochondrial oxidation of SFAs and promoting their accumulation in the form of triacylglycerol (TAG) (30).

High levels of FFAs are associated with insulin resistance and can even lead to it. As early as 1963, Randle et al. suggested a correlation between elevated blood FFAs and insulin resistance, proposing the glucose-fatty acid cycle theory. They demonstrated that the oxidation of blood FFAs increases the acetyl CoA content in muscles, and the allosteric inhibition of pyruvate dehydrogenase activity by acetyl CoA reduces glucose oxidation (31). Subsequent research has shown that the aforementioned lipotoxic effects caused by excessive lipid production can directly affect intracellular signaling pathways. For example, in the gastrocnemius muscle of rats fed a high-fat diet, the level of PKC increased, leading to a weakening of insulin signaling (32). A recent clinical case-control study found that total FFA and SFA increased the homeostatic model assessment of insulin resistance (HOMA-IR), thereby increasing the risk of developing T2DM (33).

### 3.2 Changes in FFA profile in T2DM

In patients with T2DM, the plasma total FFA level is elevated (34, 35). The most commonly observed pattern in T2DM patients is the increase in SFAs, especially C16:0 (21). Impaired insulin secretion, reduced insulin sensitivity, and poor glucose tolerance are closely related to the increased plasma FFA level, particularly SFAs, including C16:0 and C18:0 (36). Additionally, in T2DM patients, the level of palmitic acid is positively correlated with the level of glycosylated hemoglobin (HbA1c), while in patients with poor diabetes control, the level of oleic acid among MUFAs is only correlated with HbA1c levels (37).

The correlation between plasma FFA and T2DM varies depending on the type of fatty acid. A large cohort study involving 95,854 participants indicates that plasma SFAs and MUFAs concentrations are associated with a higher risk of T2DM, while plasma omega-3 PUFA and omega-6 PUFA are associated with a lower risk (38). Even within the same category of fatty acids, such as SFAs or UFAs, the correlation of specific individual fatty acids with T2DM can differ. Systematic reviews and large cohort studies indicate that an increase in odd-chain SFAs is associated with a reduced risk of T2DM events, while an increase in even-chain SFAs is associated with an increased risk of T2DM events (39, 40). A large-scale study measuring plasma phospholipid PUFA in 12,132 new cases of T2DM and 15,919 control participants indicated a significant negative correlation with plant-derived omega-3 PUFA (alpha-linolenic acid, ALA), while no significant association was found between marine-derived omega-3 PUFAs (EPA and DHA) and T2DM (41), although there are opposing viewpoints (42). According to a recent meta-analysis of prospective cohort studies, high dietary intake of linoleic acid (LA, 18:2n-6) and increased levels of LA in the body are significantly associated with a reduced risk of T2DM (43).

## 4 FFA profile and T2DM complicated by coronary heart disease

### 4.1 Mechanisms of FFA profile inducing atherosclerosis

Endothelial dysfunction (ED) is a key event in the pathogenesis of diabetes-related atherosclerosis and vascular complications (44). Excessive FFA have been shown to lead to endothelial dysfunction, vascular hypertrophy, and vascular wall stiffness, all of which are important triggers for hypertension and atherosclerosis (45). FFA-mediated endothelial dysfunction involves multiple mechanisms, including impaired insulin signaling and nitric oxide production, oxidative stress, inflammation, activation of the renin-angiotensin system, and endothelial cell apoptosis (46).

NO is a vasodilator released by endothelial cells, and impaired production of NO is a major characteristic of ED. Elevated FFA promote insulin resistance, inhibiting the insulin signaling pathway PI3K/Akt, which leads to a reduction in NO production regulated by this pathway (46). High concentrations of FFA cause dysfunction in the electron transport chain of mitochondria, increasing the generation of reactive oxygen species (ROS) and inducing oxidative stress in the body, which can lead to apoptosis of endothelial cells (47). In healthy subjects, FFA-induced vascular risk factors can be observed, characterized by elevated levels of endothelial markers (18). Additionally, FFA also induces inflammation through the activation of NF-κB (48).

**Figure 1:**
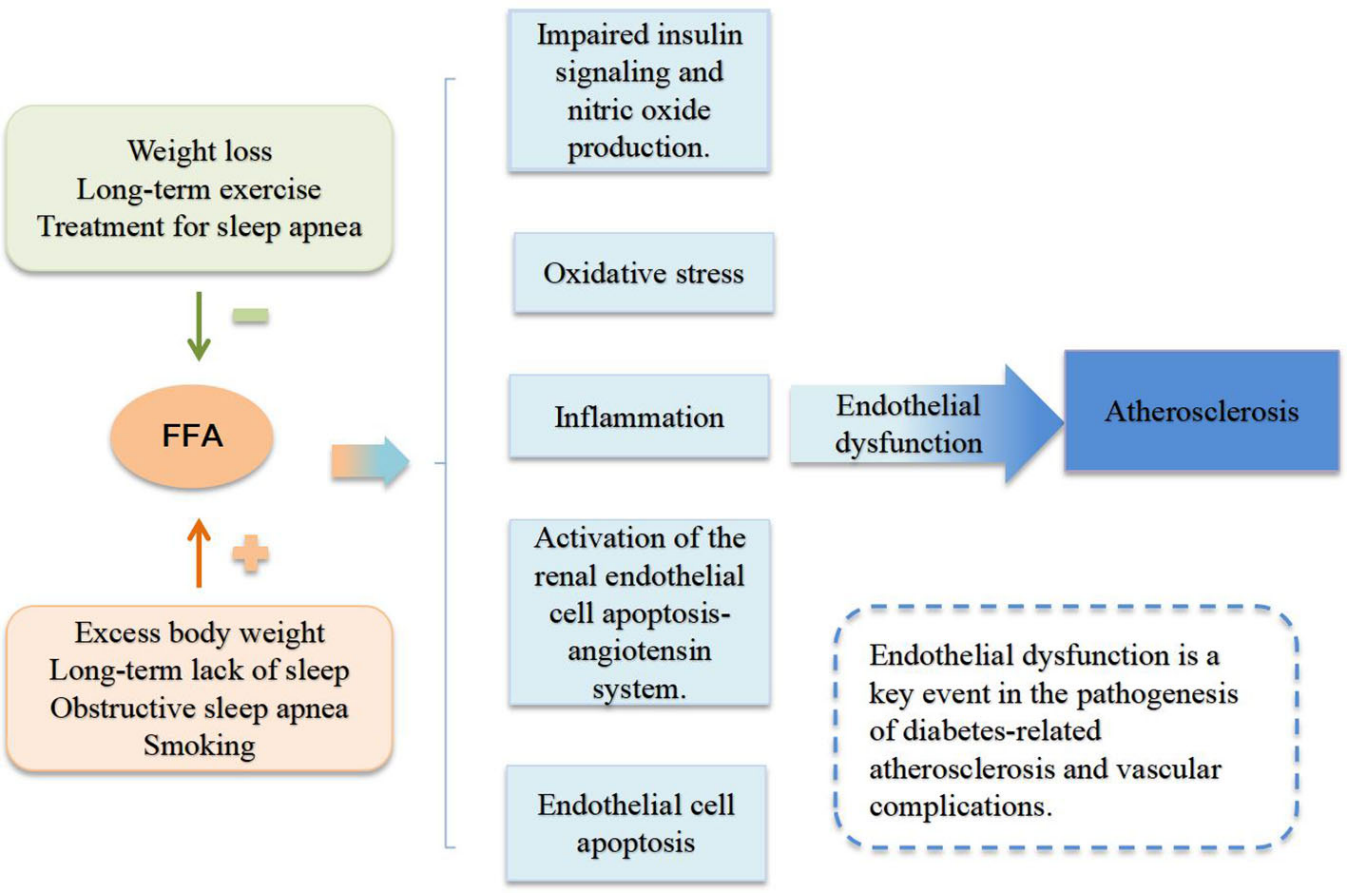
The mechanisms by which FFA induce atherosclerosis and the factors influencing FFA level.

Several factors can affect plasma FFA level; for instance, intense physical activity or emotional fluctuations under physiological conditions can temporarily elevate FFA level, which is a natural response of the body to meet energy demands. Additionally, certain lifestyle and clinical factors, such as obesity, chronic sleep deprivation, sleep apnea, and smoking, can increase FFA level. Conversely, weight loss, regular exercise, and treatment of sleep apnea can lower FFA level, potentially alleviating the adverse effects caused by elevated FFA (49). **Figure 1** summarizes the mechanisms by which FFA induce atherosclerosis and the factors influencing FFA level.

Researchers believe that long-chain omega-3 fatty acids are beneficial for cardiovascular health, including EPA and DHA, which mainly come from oily fish, as well as alpha-linolenic acid (ALA), which mainly comes from plants (50). Increased consumption of EPA and DHA, or elevated levels of them, is linked to a reduced risk of cardiovascular diseases, especially coronary heart disease, and lower rates of cardiovascular-related deaths (51–54).

### 4.2 The role of FFA profile in T2DM complicated by coronary heart disease

In the population with T2DM, previous studies have focused more on the relationship between the total level of FFA and cardiovascular diseases, while research on FFA profiles has been limited. In **Table 1**, we list the characteristics of studies and survey populations regarding FFA and FFA profiles, while specific changes in FFA profiles are detailed in **Table 2**. Total FFA level is considered a risk factor for the development of coronary heart disease and other arterial vascular lesions in T2DM, with previous research results being consistent (15, 19, 56, 58–61). In addition, high level of FFA indicate a poor prognosis for coronary heart disease (20), suggesting a more severe condition (16, 19, 61). The findings concerning FFA profiles differ, which can be attributed to some factors, such as the ethnic backgrounds of the study groups, differences in the chosen control groups, the necessity of fasting prior to blood sampling, and whether the results are shown as absolute concentrations or as a percentage of the total plasma FFA measured.

**Table 1:** Studies on population characteristics and blood concentrations of individual FFA species in cardiovascular subjects and controls of patients with T2DM.

	Country	Year	Number of subjects (Control vs Case)	Match or not^+^	Affected blood vessels	Case group definition	Research Conclusion	Sample type
The total level of FFA	(15) India	1995	164:213	Unmatched	Coronary	-	Higher FFA levels seem to be a predictor of CHD in diabetes patients, exceeding the effect of abnormal lipoproteins.	Plasma
	(55) Sweden	2000	140:343	Unmatched	Coronary, cerebrovascular	-	High NEFA concentrations in healthy offspring with a family history of diabetes were significantly associated with their parents’ myocardial infarction and stroke.	Serum
	(16) Japan	2002	23:51	Age-matched	Coronary	Medial thickness of intima without plaque segment [IMT]	Serum NEFA levels independently predict the extent of plaque segmental stenosis in diabetes patients, but not in diabetes subjects.	Serum
	(56) Canada	2002	99:103	Age, body mass index, smoking habits and alcohol intake-matched	Coronary	Diagnostic electrocardiogram (ECG) changes or meets the following two criteria: Typical chest pain lasts for at least 20 minutes, Abnormal creatine kinase levels should be at least twice the upper limit of normal Typical chest pain lasts for at least 20 minutes, with abnormal creatine kinase levels at least twice the upper limit of normal.	Elevated plasma FFA concentration is associated with increased risk of ischemic heart disease.	Plasma
	(17) Spain	2010	20:20	Unmatched	Carotid artery	-	The presence of NEFA in the new intima of diabetes plaque increased. The level of NEFA in atherosclerotic plaque of diabetes was higher than that of non-diabetes subjects. NEFA may occur locally and lead to local inflammation.	serum and plaque
	(57) China	2011	51:34	Unmatched	Coronary	coronary angiography	The levels of FFA and hs CRP in serum are closely related to type 2 diabetes and diabetes with coronary atherosclerotic heart disease.	Serum
	(58) China	2014	103:59	Unmatched	Coronary	CTnT ≥ 0.01 ng/L+other evidence	There is a positive correlation between serum FFA levels and HDL levels in T2DM and the control group.	Serum
	(59) China	2016	45:50	Unmatched	Coronary	Coronary CT scan	T2DM combined with CHD coronary plaques are mainly soft plaques and mixed plaques, with extensive coronary artery lesions. Carotid ultrasound shows that the more peripheral vascular plaques and the more branches of coronary artery lesions, the more severe the lesions. Combining CCTA, carotid ultrasound, hsC-RP, and FFA levels in clinical practice can improve the diagnosis rate of T2DM complicated with CHD.	Serum
	(60) China	2017	79:84	Unmatched	Coronary	coronary angiography	FFA, Hcy, CRP, FPG are positively correlated with coronary heart disease in elderly patients with type 2 diabetes.	Serum
	(18) America	2018	40	-	-		The increase in plasma FFA within the physiological range observed in obesity and T2DM induces endothelial activation, vascular inflammation, and thrombosis markers in healthy subjects.	Plasma
	(20) China	2019	1039:1606	Unmatched	Coronary	coronary angiography	Baseline FFA levels are associated with prognosis in patients with DM and pre DM CAD.	Serum
	(19) China	2020	70:232	Unmatched	Coronary, carotid artery	coronary angiography, Carotid ultrasound	Elevated FFA levels seem to be associated with the presence and severity of CAD and CAP in T2DM patients.	Plasma
	(61) China	2021	84:80	Unmatched	Coronary	coronary angiography	The serum FFA level is independently correlated with severe coronary artery calcification and may have a certain predictive ability for severe coronary artery calcification. The DM group has the strongest correlation.	Serum
FFA profile	(62) China	2011	55:55	Age and sex-matched	Carotid artery, femoral artery, internal iliac artery	Carotid artery intima-media thickness, femoral artery intima-media thickness, iliac artery intima-media thickness Using high-resolution color ultrasound measurement, Patients with IMT>1.0mm in any part and/or plaques in the field of view without clinical manifestations Defined as subclinical atherosclerosis (subclinical atherosclerosis)	Saturated fatty acids (C16:0, C18:0), monounsaturated fatty acids (C18:1) Total fatty acid (TFA) is associated with subclinical atherosclerosis in type 2 diabetes and is the most common Significant influencing factors.	Plasma
	(63) China	2016	36:137	Unmatched	Coronary, cerebrovascular, peripheral vascular	Clinical diagnosis includes coronary heart disease, cerebrovascular disease, peripheral vascular disease, and hypertension	Comparison of fatty acid composition between large vessel disease groups: TFA, MUFA, SFA, and C16:0, C18:0, C18:1 significantly increased with the severity of vascular disease.	Serum
	(64) Japan	2017	533:144	Unmatched	Coronary	Pathological Q waves are observed on the electrocardiogram, and there are areas of viable myocardial loss detected on imaging	PMI patients with DM have lower levels of EPA/AA and DHA/AA. The use of statins may affect DHA/AA, but not EPA/AA, so the EPA/AA ratio is a better biomarker for evaluating cardiovascular events.	Serum
	(65) China	2021	35:68	Unmatched	Carotid artery	Carotid artery ultrasound shows that the intima-media thickness (IMT) f 1.2mm/IMT ≥ 1.5mm and/or the formation of atherosclerotic plaques are consistent	1. The composition of blood free fatty acid profiles in patients with T2DM combined with AS and those with T2DM alone In terms of differences, the levels of C16:0 and C18:0 were significantly higher in patients with T2DM alone. 2. C16:0 is the main influencing factor of atherosclerosis in type 2 diabetes patients, Significantly increase the risk of atherosclerosis.	Serum
	(66) China	2022	134:126	Unmatched	Coronary	Acute myocardial infarction (MI), asymptomatic myocardial infarction, or previous coronary artery surgery	Multivariate logistic regression analysis showed that after adjusting for confounding factors and other risk factors, dozens of FFAs were independent risk factors for CHD.	Plasma

**^+^** Match refers to the consistency of certain characteristics between the case group and the control group.

**Table 2:** Changes in the concentration of FFAs in the blood of cardiovascular subjects and controls of patients with T2DM.

Types of FFA	Changes in plasma free fatty acid concentrations in cardiovascular subjects compared with controls				
	(66)	(64)	(65)	(63)	(62)
Total FFA	↑			↑	↑
Saturated FFA	↑			↑	
C14:0, myristic acid	↑				
C15:0,pentadecanoic acid					
C16:0, palmitic acid	↑		↑	↑	↑
C17:0,heptadecanoic acid	↑				
C18:0, stearic acid	↑		↑	↑	↑
C19:0, nonadecylic acid					
C20:0, arachidic acid	↑				
C22:0, behenic acid	↑				
C24:0, lignoceric acid					
Monounsaturated FFA	↑			↑	
C14:1n-9,myristoleic acid					
C16:1n-7,palmitoleic acid	↑				
C16:1n-9,cis-7 hexadecenoic acid	↑				
C17:1(cis-10), cis-10-heptadecenoic acid	↑				
C18:1n-9, oleic acid (OA)	↑			↑	↑
C18:1trans-n-7, vaccenic acid	↑				
C19:1n-9,cis-10 nonadecenoic acid					
C20:1n-9, gondoic acid	↑				
Polyunsaturated FFA					
C16:2cis-9,12-hexadecadienoic acid					
PUFA n-3	↑				
C18:3n-3,α-linolenic acid (ALA)					
C20:5n-3, eicosapentaenoic acid (EPA)	↑	↓			
C22:3n-3 docosatrienoic acid	↑				
C22:5n-3, docosapentaenoic acid	↑				
C22:6n-3, docosahexaenoic acid (DHA)	↑	↓			
PUFA n-6	↑				
C18:2n-6, linoleic acid (LA)					
C18:3n-6,γ-linolenic acid (GLA)					
C20:2n-6, eicosadienoic acid	↑				
C20:3n-6, dihomo-γ-linolenic acid (DGLA)	↑				
C20:4n-6, arachidonic acid (ARA)	↑				
C22:4n-6, adrenic acid	↑				

Use ↑ to indicate an increase in the concentration of FFAs and ↓ to indicate a decrease.

In T2DM, the levels of C16:0 and C18:0 in the blood are significantly increased in patients with concurrent cardiovascular disease, as noted in multiple studies (62, 63, 65, 66). This is consistent with previous research, as C16:0, the most abundant SFA in the human body, is associated with an increased cardiovascular risk in T2DM patients (67). A 2017 multicenter cross-sectional study from Japan only assessed the association between PUFA levels and risk factors in patients with prior myocardial infarction, finding that the levels of C20:5 (EPA) and C22:6 (DHA) were lower in the DM group with coronary heart disease, which aligns with findings from numerous previous studies (68). Additionally, this study had a larger sample size compared to four others and excluded subjects taking omega-3 PUFA supplements, which may have led to more accurate measurements. The research conducted by Hu et al. analyzed the plasma FFA profile of 134 patients with T2DM and 126 patients with T2DM complicated by coronary heart disease (CHD). The results indicated that nearly all types of FFAs, regardless of their carbon chain length or unsaturation, were significantly elevated in the plasma of patients with CHD-T2DM compared to those with uncomplicated T2DM. Additionally, the study highlighted differences in the levels of omega-3 fatty acids, specifically C20:5 (EPA) and C22:6 (DHA), when compared to earlier research findings (66). The reasons for the differences may be related to the discrepancies found in previous randomized controlled trials. Although numerous epidemiological and clinical studies have indicated that supplementation with Omega-3 PUFAs is associated with reduced inflammation, the levels of Omega-3 PUFAs are generally lower in patients with cardiovascular diseases (68). However, there are also large randomized controlled trials that show that supplementation with Omega-3 fatty acids does not reduce the incidence of cardiovascular events in high-risk patients with T2DM (69). Based on the above research, the changes in the concentrations of FFAs associated with T2DM are complex and require further studies.

### 4.3 The relationship between dietary intake of fatty acids and T2DM complicated by coronary heart disease

As early as the 1970s, epidemiological studies of Greenland Eskimos linked their diet, rich in omega-3 PUFAs from fish and fish oil, to low cardiovascular mortality and effective treatment of hypertriglyceridemia (70). Subsequently, the health benefits of fish oil and its content of omega-3 long-chain PUFAs (omega-3 LC-PUFAs) have garnered widespread attention in the scientific community over the past four decades. Fish oil contains high levels of but variable omega-3 LC-PUFAs, including EPA and DHA, as well as small amounts of docosapentaenoic acid (DPA; 22:5n-3) (71). However, the use of omega-3 PUFAs for the prevention of coronary heart disease remains controversial. The data results from classic large-scale RCT studies show that, supplementation with larger doses of pure EPA demonstrates cardiovascular benefits (72, 73), while supplementation with a mixture of EPA and DHA yields negative results (74, 75). The types of fish oil prescription drugs (pure EPA or EPA+DHA mixture) and dosage differences may be important factors affecting clinical outcomes. In addition to marine derived omega-3 LC-PUFA, plants synthesize short chain omega-3 fatty acids such as alpha linolenic acid (ALA; 18:3, n-3) and linoleic acid (18:2, n-6), which are present in many seeds, nuts, and seed oils (76). The sustained effects of ALA intake on cardiovascular health also exhibit heterogeneity (50, 77), which may be related to the dosage used (78).

**Figure 2:**
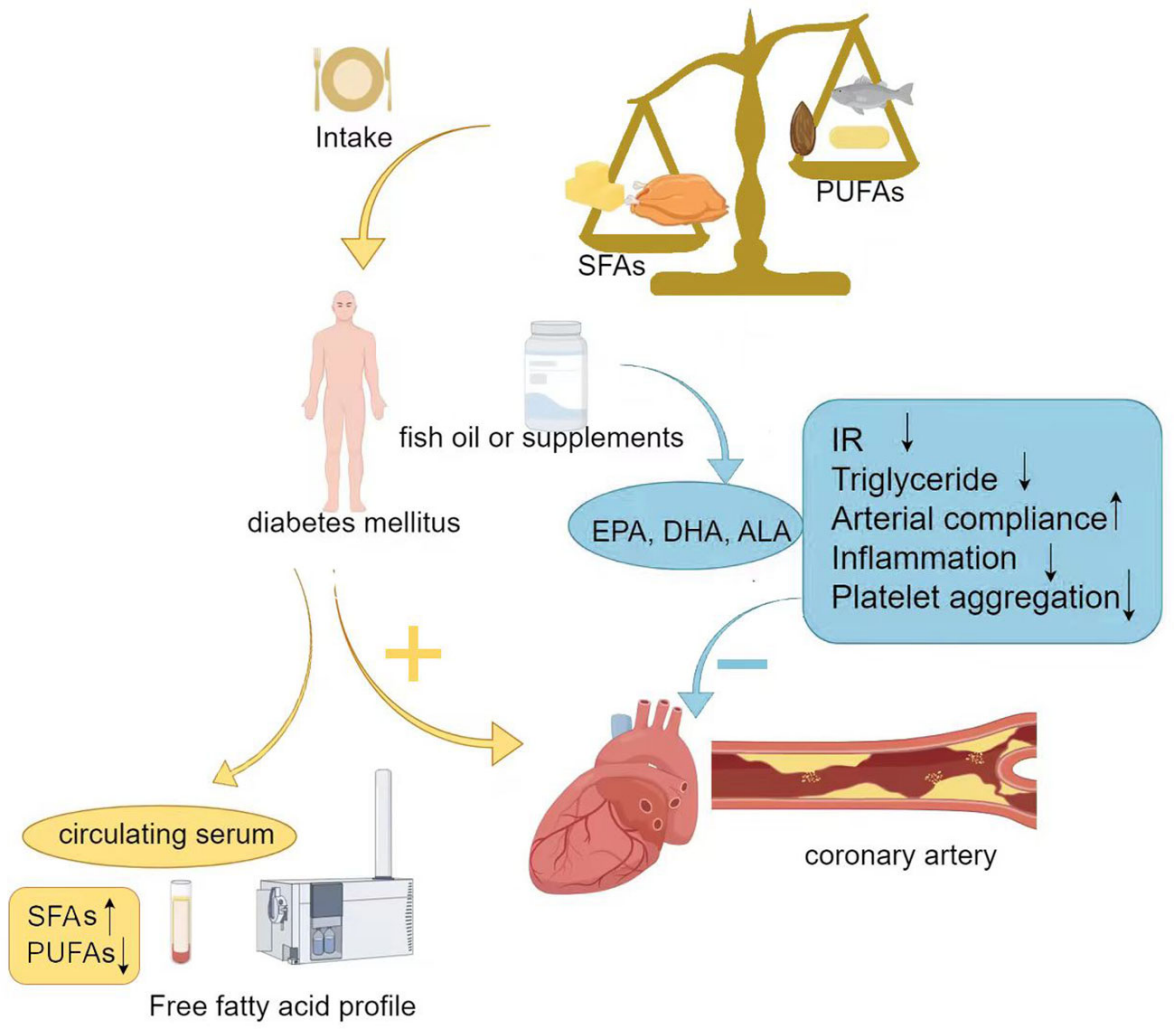
Association between dietary intake of fatty acids and T2DM with coronary heart disease. SFAs, Saturated fatty acids; PUFAs, Polyunsaturated fatty acids; DHA, Docosahexaenoic acid; EPA, Eicosapentaenoic acid; ALA, α-Linolenic acid; IR, Insulin resistance.

Multiple large systematic reviews indicate that the Mediterranean diet, which is rich in PUFAs and MUFAs, can improve blood glucose and lipid levels in patients with T2DM (79, 80). However, the evidence does not support the recommendation for all diabetes patients to take EPA and DHA supplements to prevent or treat cardiovascular events. In the ASCEND trial (81), supplementation with omega-3 fatty acids (EPA, DHA) at a dose of 1 g/day did not provide cardiovascular benefits compared to placebo in diabetic patients without evidence of cardiovascular disease. However, the results of the REDUCE-IT study found that supplementation with 4 g/day of pure EPA significantly reduced the risk of adverse cardiovascular events. This trial involved 8,179 participants, more than 50% of whom had diabetes. In patients with cardiovascular disease who were receiving statin treatment, had achieved low-density lipoprotein cholesterol targets, and had elevated triglyceride levels (135-499 mg/dL), there was an absolute reduction of 5% in cardiovascular events (82). **Figure 2** shows the association between dietary intake of fatty acids and T2DM with coronary heart disease.

Many countries’ dietary guidelines advocate for limiting the intake of SFAs, especially for individuals with clinical atherosclerotic cardiovascular disease (ASCVD), dyslipidemia, or diabetes, such as in China, Europe, and the United States (83–86). The results of observational studies indicate that dietary patterns with lower average SFA intake are associated with good cardiovascular outcomes (87). In addition, although the number of randomized controlled trials testing the impact of reducing SFAs intake on ASCVD outcomes is limited, existing evidence supports the view that replacing SFAs with UFAs, especially PUFAs, may reduce the risk of ASCVD (88). In addition to increasing the concentration of LDL-C and atherogenic lipoprotein particles, higher SFAs intake may also affect the pathways that affect inflammation, rhythm, hemostasis, apolipoprotein CIII production and high-density lipoprotein function (87). However, the impact of these effects on ASCVD risk remains uncertain.

As the most abundant saturated fatty acid in the human body, C16:0 has a higher content in palm oil, animal fat, and cocoa butter. A recent study that combines human cohort studies and animal models has identified key mechanistic pathways through which C16:0 reduces plaque stability, which is associated with increased cardiovascular risk in patients with T2DM (67). And C18:1 oleic acid, one of the MUFAs, may have a protective effect on T2DM (89), while also reducing the proportion of low-density lipoprotein cholesterol and total cholesterol, and lowering cardiovascular risk. C18:1 reduces insulin resistance by increasing the mitochondrial oxidation of SFAs and promoting their accumulation in the form of TAG, thereby decreasing the synthesis of DAG and ceramides (22).

Currently, there is no unified conclusion regarding the optimal daily total fat energy ratio for patients with T2DM. The 2020 CDS guidelines recommend that the energy provided by fat in the diet should account for 20% to 30% of total energy (90). If the fat is of high quality (such as MUFAs and ω-3 PUFAs), the fat energy ratio can be increased to 35%. It is advised to limit the intake of SFAs and trans fatty acids as much as possible.

### 4.4 The relationship between obesity and T2DM, coronary heart disease

Obesity is a chronic positive energy balance disease caused by a complex interaction between abnormal neurohumoral responses and an individual’s socioeconomic, environmental, behavioral, and genetic factors, leading to a state of chronic inflammation. The World Health Organization defines individuals with a body mass index (BMI) >25 kg/m² as overweight and those with a BMI >30 kg/m² as obese. Obesity directly or indirectly increases the incidence and mortality of cardiovascular disease (CVD). The direct effects mainly include two aspects: one is the structural and functional adaptations of the cardiovascular system induced by obesity (91). The second is that adipocytes synthesize and secrete a large number of proteins and hormones known as adipokines, which play important roles in endocrine regulation, immune function, and inflammation. In obesity, adipose tissue undergoes maladaptive expansion, leading to impaired secretion of adipokines and an imbalance of adipokines. There is an increase in pro-inflammatory adipokines and a decrease in anti-inflammatory adipokines, resulting in the development of a chronic, low-grade inflammatory state that negatively impacts vascular homeostasis (92). Additionally, abnormal fat deposition, epicardial adipose tissue, and non-alcoholic fatty liver disease are also pathophysiological factors through which obesity leads to or exacerbates cardiovascular diseases (93). The indirect effects are that obesity directly leads to the development of diabetes, hypertension, and hyperlipidemia, which are also risk factors for CVD.

**Figure 3:**
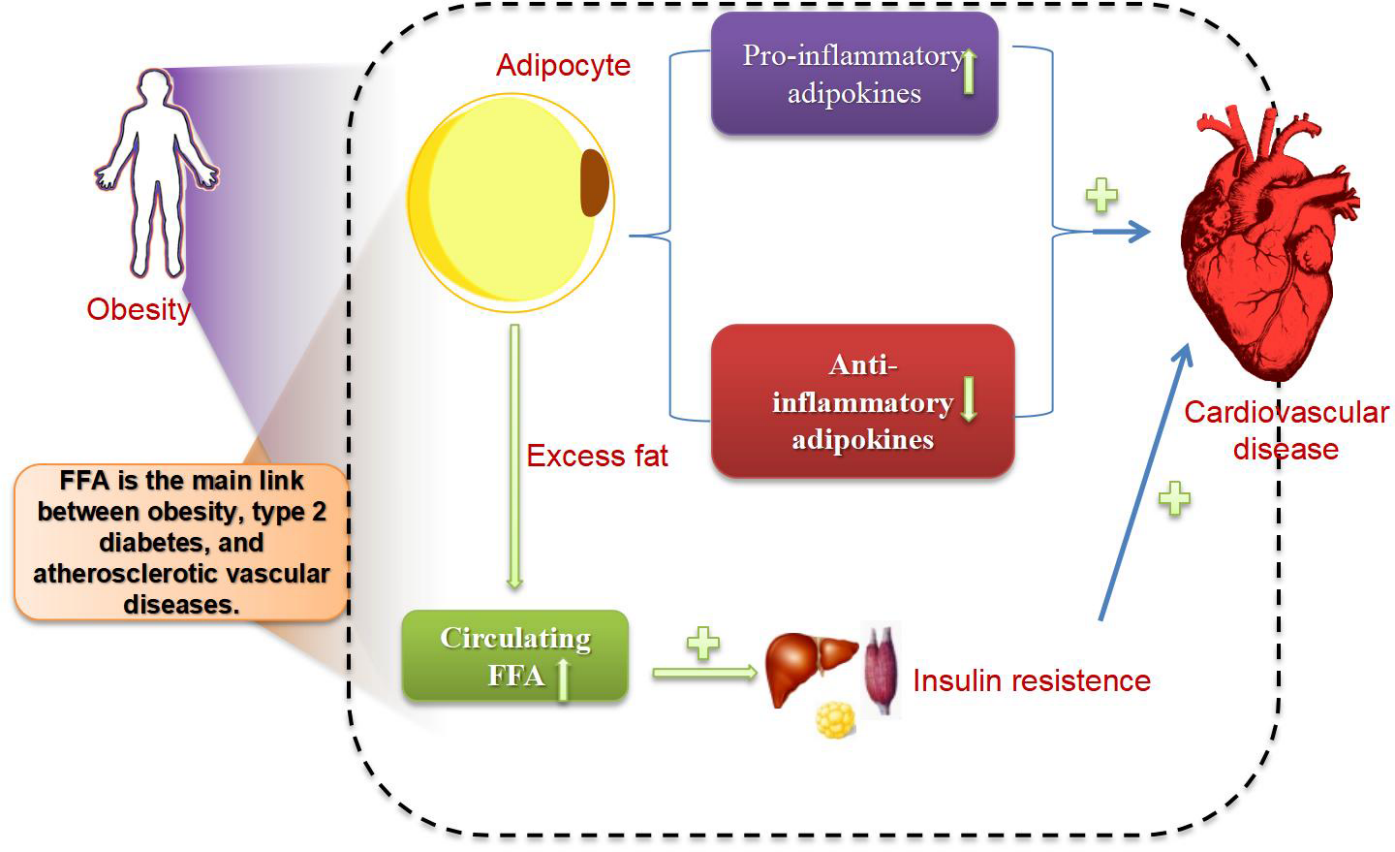
The relationship between obesity, T2DM, and coronary heart disease.

Obese patients who consume excessive calories for a long time lead to an excess storage of fat, surpassing the limited storage capacity of adipose tissue for fatty acids, which results in an increase in circulating FFAs. The lipotoxicity of fatty acids in circulation and their storage in key metabolic regulatory organs leads to systemic oxidative stress, inflammation, and metabolic dysregulation. FFA can cause insulin resistance in all major insulin target organs (skeletal muscle, liver, endothelial cells) (94) and has become a major link between obesity, T2DM, and atherosclerotic vascular diseases. **Figure 3** illustrates the connection between them.

### 4.5 The relationship among statins, FFA, type 2 diabetes, and coronary heart disease

Elevated plasma FFA level associated with T2DM can lead to various pathologies, making it clinically important to control their concentrations. In addition to antidiabetic medications, lipid-lowering drugs are also used for the management of T2DM. However, most of these lipid-lowering drugs primarily control plasma cholesterol, LDL, and HDL levels rather than FFA level. There is evidence that statins (used for coronary artery disease) have anti-inflammatory effects and may also impact FFA level, potentially reducing plasma FFA concentrations (95).

Dyslipidemia in diabetes is characterized by elevated fasting and postprandial triglycerides, low levels of high-density lipoprotein cholesterol, elevated low-density lipoprotein cholesterol, and a predominance of small, dense low-density lipoprotein particles. Lifestyle changes and blood glucose control may improve lipid levels, but lowering LDL is the cornerstone of treating diabetic dyslipidemia, with statins being the primary therapeutic agents (96). They have beneficial effects on dyslipidemia associated with coronary artery disease (97). Therefore, most diabetic patients should receive statin therapy (98). However, it is worth noting that statins seem to have negative effects on non-diabetic patients, with a higher risk of developing new-onset T2DM (99).

## 5 The potential of FFA as biomarkers

Changes in FFA are more sensitive than changes in triglycerides and cholesterol esters in reflecting the body’s lipid metabolism (100). Circulating FFA levels are closely related to insulin sensitivity and the decline of β-cell function (101). Serum FFA levels are useful biomarkers for improving the management of patients with T2DM, as they can reflect the severity of coronary artery disease (CAD) and carotid atherosclerotic plaques (CAP) in T2DM patients (19). Additionally, FFA is closely associated with metabolic syndrome (MS) and is a predictor of non-alcoholic fatty liver disease (NAFLD) (102). In patients with high fasting blood glucose, a decrease in arachidonic acid (AA) and an increase in the EPA/AA ratio have been observed. This suggests that they have the potential to serve as biomarkers for blood glucose control (103).

## 6 Conclusion and future directions

Elevated levels of FFAs in plasma are associated with the presence and severity of cardiovascular events in patients with T2DM. However, previous studies have primarily focused on the total FFA level in plasma and the effects of dietary interventions, with little research on the levels of various FFA components in the FFA profile. Exploring the FFA profile is of great value for further research into the metabolic mechanisms of coronary heart disease and diabetes, predicting disease risk, and guiding prognosis and interventions. However, coronary heart disease combined with diabetes is very complex, and assessing the blood FFA profile alone cannot provide a complete picture of individual risk; it must also consider diet, lifestyle, and other factors. Therefore, exploration of the FFA profile should continue, with the hope of achieving more effective translation and application in clinical practice.
